# The correlation between severe asymptomatic carotid artery stenosis and severe multi-organ dysfunction after off-pump coronary artery bypass grafting

**DOI:** 10.3389/fcvm.2024.1399727

**Published:** 2024-11-19

**Authors:** Tong Wang, Chang Zhao, Jian Cao, Kui Zhang, Rui Wang, Yu Xiao, Ran Dong, Jiayang Wang

**Affiliations:** ^1^ Department of Cardiac Surgery, Beijing AnZhen Hospital, Capital Medical University, Beijing, China; ^2^ Department of Ultrasound, Beijing Anzhen Hospital, Capital Medical University, Beijing, China; ^3^ Department of Radiology, Beijing Anzhen Hospital, Capital Medical University, Beijing, China

**Keywords:** severe asymptomatic carotid artery stenosis, off-pump coronary artery bypass grafting, severe multi-organ dysfunction, Sequential Organ Failure Assessment, 30-day postoperative mortality, stroke

## Abstract

**Background:**

The current research aimed to demonstrate the independent association between preoperative severe asymptomatic carotid artery stenosis (ACAS) and severe multi-organ dysfunction after off-pump coronary artery bypass grafting (OPCAB), which may further indicate the relationship between severe ACAS and adverse 30-day postoperative outcomes of patients undergoing OPCAB.

**Methods:**

This was a single-center, retrospective observational study including patients without a history of stroke or Transient Ischemic Attacks (TIA) (asymptomatic), who underwent for an isolated OPCAB in the center for operative treatment of coronary artery disease of Beijing Anzhen Hospital from January 2020 to December 2021. All enrolled patients underwent carotid artery ultrasound prior to OPCAB. The information was extracted independently by two authors of the study from the medical records. Both univariate and multivariate analyses were conducted.

**Results:**

A total of 562 patients met the inclusion criteria for the current study. 63 (11.2%) suffered from severe ACAS. The Sequential Organ Failure Assessment (SOFA) maximum in the severe ACAS group was significantly higher than that in the non-severe ACAS group (9.76 ± 3.03 vs. 7.75 ± 2.96, *p* < 0.0001), and a higher proportion of patients in the severe ACAS group exhibited severe multi-organ dysfunction (44.4% vs. 14.0%, *p* < 0.0001). In addition, severe ACAS was related to an increased rate of 30-day postoperative major adverse cardiovascular and cerebral events (MACCEs), including a 30-day postoperative stroke. Severe ACAS was associated with an elevated risk of delirium, and acute kidney injury (AKI). The results of the multivariate analysis demonstrated that severe ACAS may be independently associated with severe multi-organ dysfunction (OR, 7.37, 95% CI 4.80–14.30, *p* < 0.0001) after OPCAB. Also, severe ACAS may be independently associated with 30-day postoperative stroke (OR, 2.83, 95% CI 1.03–7.75, *p* = 0,043).

**Conclusions:**

Severe ACAS was independently associated with severe multi-organ dysfunction after OPCAB, which may be associated further with an increased rate of 30-day postoperative mortality and complications. This study highlights: (1) the importance of personalized assessment for potential advantages and disadvantages in prognosis of severe ACAS patients undergoing OPCAB with carotid endarterectomy; (2) the role of multi-organ parameters, especially cardio-cerebral factors, should be emphasized during the process of severe ACAS management.

## Introduction

The internal carotid artery ascends to the base of the skull, traverses the petrous portion of the temporal bone, and gains entry into the cranial cavity through the foramina. And carotid artery disease typically refers to a range of conditions characterized by structural changes in the lumen of the carotid artery. The optimal management for coronary artery disease patients undergoing off-pump coronary artery bypass grafting (OPCAB), combined with carotid artery disease has been regarded as the “bottleneck” problem, requiring urgent resolution in the field of cardiac surgery (1–5). A conclusive advantage from carotid endarterectomy in mitigating stroke risks has been demonstrated for severe symptomatic carotid artery disease (6–8). However, a controversy persists regarding the management of severe asymptomatic carotid artery stenosis (ACAS) (9) and no definitive conclusions have been reached in the current guidelines for the optimal surgical management of patients with severe ACAS and coronary artery disease (CAD) (2, 10–12) (Table 1). In addition, the level of the evidence in the above guidelines is low. The root cause of the disputed conclusions for the optimal management of severe ACAS and CAD patients undergoing OPCAB, we thought that the relationship between severe ACAS and adverse 30-day postoperative outcomes, particularly the 30-day postoperative mortality in surgical patients, has not been determined by previous clinical data.

**Table 1:** Review of current guidelines for the surgical management of patients with ACAS and coronary artery disease.

Guidelines	Luminal narrowing	Routine carotid revascularization	Class of recommendation	Level of evidence
Canadian cardiovascular society 2022 guidelines (2)	NA	Against	Weak	Low-Quality
ESC/ESVS 2017 (10)	(a). 70%–99% (b). Bilateral 70%–99% CAS or 70%–99% CAS and contralateral occlusion (Severe ACAS) (c). 70%–99% CAS in the presence of risk factors for ipsilateral stroke (Severe ACAS)	Against Against	III IIb	B B
		Recommend	IIb	C
ACCF/AHA 2011 (11)	Bilateral 70% to 99% CAS or a unilateral 70% to 99% CAS with a contralateral occlusion (Severe ACAS)	Recommend	IIb	C
ASA…SVS 2011 (12)	NA	Against	IIb	C

ACAS, asymptomatic severe carotid artery stenosis.

In addition, the Sequential Organ Failure Assessment (SOFA) maximum was reported to be the gold standard for the prognostic evaluation of OPCAB (13–15), and the severe multi-organ dysfunction, measured by SOFA maximum ≥11, has been confirmed to predict the 30-day postoperative mortality in patients undergoing isolated OPCAB (13). Since the independent correlation between severe ACAS and 30-day postoperative mortality in patients undergoing OPCAB cannot be explored directly, it is limited by sample size. Therefore, it is imperative to advance the study endpoint by prioritizing severe multi-organ dysfunction over the 30-day postoperative mortality as the primary endpoint of our current research.

To catch the above knowledge gap, the current research aimed to demonstrate the independent association between preoperative severe ACAS and severe multi-organ dysfunction after OPCAB, which may further indicate the relationship between severe ACAS and adverse 30-day postoperative outcomes of patients undergoing OPCAB.

## Methods

The Ethics Committees of Beijing Anzhen Hospital, Capital Medical University (Beijing, China), approved the current observational research. Ethics number: 2024205x.

### Patients’ selection

**Figure 1:**
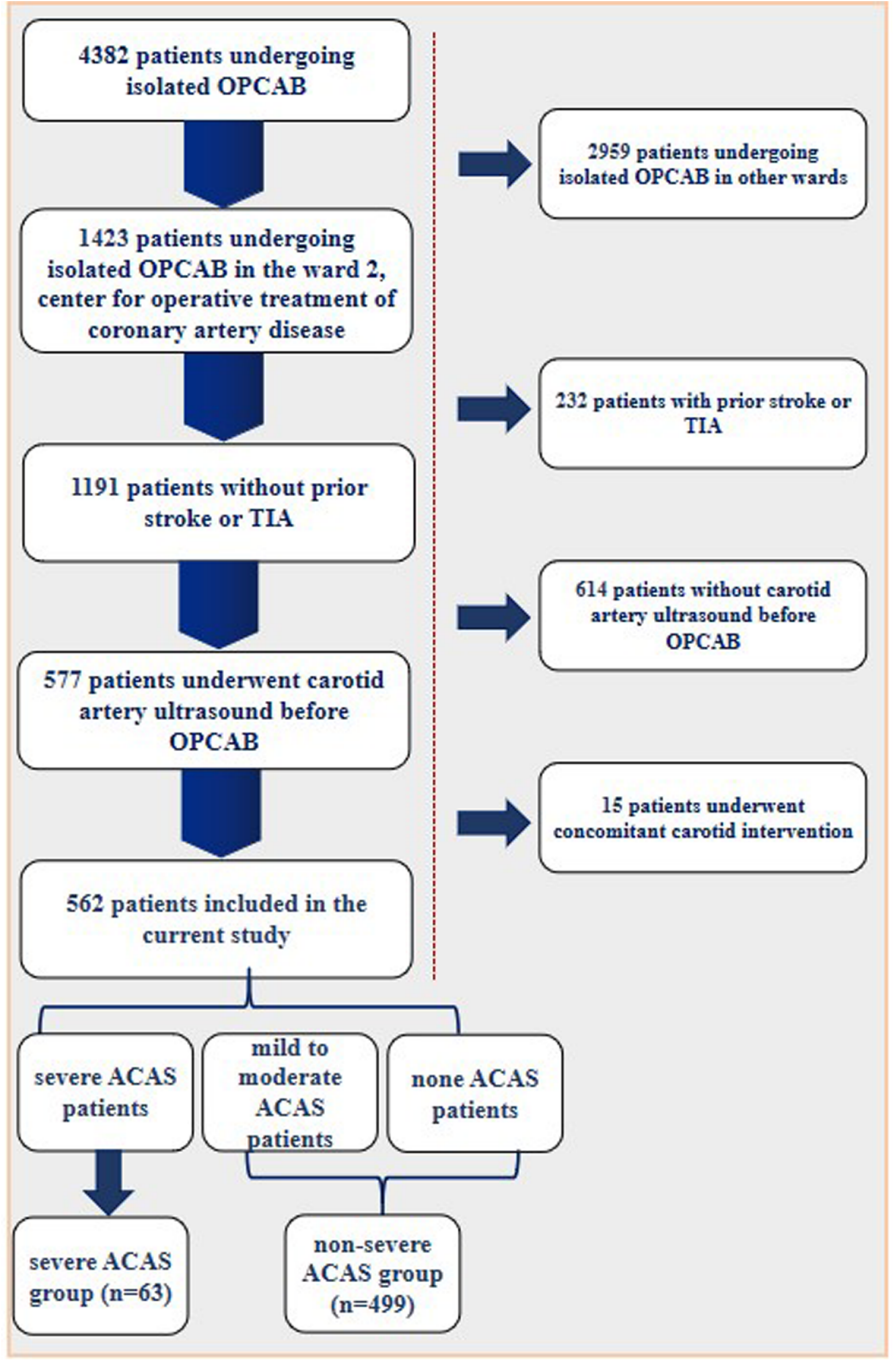
Patient enrollment flow diagram.

This study included 562 patients without a history of stroke or Transient Ischemic Attacks (TIA) (asymptomatic), who underwent for an isolated OPCAB in the ward 2, center for operative treatment of coronary artery disease of Beijing Anzhen Hospital from January 2020 to December 2021. Patients with prior stroke or TIA, with previous or recent carotid intervention, undergoing other cardiac surgeries, such as aortic or valve operations, undergoing OPCAB combined with above surgeries were all excluded. All enrolled patients underwent carotid artery ultrasound prior to OPCAB. The specific enrollment of patients was shown in Figure 1. The information was extracted independently by two authors of the study from the medical records. Informed consent was obtained from the patient or their relatives on the day of admission.

### Definitions, patients’ grouping, and study endpoints

Based on the latest guidelines for the carotid arterial disease (2, 10–12). The severity of CAS was defined according to the percentage of carotid arterial luminal stenosis, with the arterial lumen distal to the stenosis as the reference diameter. Severe CAS was defined as the presence of one or more of the following: (1) bilateral 70%–99% CAS; (2) unilateral 70%–99% CAS and contralateral occlusion; and (3) unilateral 70%–99% CAS in the presence of risk factors for ipsilateral stroke or TIA. The included asymptomatic individuals were divided into two groups according to the presence or absence of severe CAS: the severe ACAS group and the non-severe ACAS group (mild to moderate ACAS and no ACAS).

The primary endpoint was postoperative severe multi-organ dysfunction, measured by SOFA score ≥11 based on the latest literature (13, 14, 16). The SOFA score (Table 2) was counted daily from the day of the surgery to the day of discharge. The SOFA maximum was reported to be the gold standard for prognostic evaluation of OPCAB. Furthermore, the secondary endpoint was the 30-day postoperative stroke, which was defined as the 30-day postoperative neurologic deficit that did not resolve within 24 h and associated with a brain lesion (Brain lesions are defined as areas of brain tissue that have been compromised due to injury or disease. These lesions are typically identified through diagnostic imaging scans, such as MRI or CT scans. They can manifest in various forms and sizes, and their presence may indicate underlying neurological conditions.).

**Table 2:** The definition of SOFA score.

Variables measurement (unit)	SOFA score				
	0	1	2	3	4
Respiratory PaO2/FiO2 (mmHg)	>400	≤400	≤300	≤200 (Values are with respiratory support)	≤100 (Values are with respiratory support)
Coagulation platelets^a^10^3 ^/μl	>150	≤150	≤100	≤50	≤20
Liver bilirubin (mg/dl)	<1.2	1.2–1.9	2.0–5.9	6.0–11.9	>12.0
Cardiovascular hypotension	None	Mean arterial pressure <70 mmHg	Dop > 5 or dob usage^a^	Dop > 5 or epi > 0.1 or norepi > 0.1^a^	Dop > 15 or epi > 0.1 or norepi > 0.1^a^
Central nervous system (Glasgow coma score/scale)	15	13–14	10–12	6–9	<6
Renal Creatinine (mg/dl) or urine output (ml/d)	<1.2	1.2–1.9	2.0–3.4	3.5–4.9 or <500	3.5–4.9 or <500

Dob, dobutamine; Dop, dopamine; Epi, epinephrine; FiO2, fraction of inspired oxygen; Norepi, norepinephrine; SOFA, sequential organ failure assessment.

^a^ Adrenergic agents administered for at least 1 h (doses given are in mg/kg per minute).

Other short-term postoperative complications include: (1) 30-day postoperative major adverse cardiovascular and cerebral events (MACCEs), which is defined as the presence of one or more of the following events: death, stroke, myocardial infarction (MI), or repeat revascularization; (2) new-onset of atrial fibrillation (AF) and ventricular arrhythmias (VA); (3) postoperative delirium; (4) pulmonary embolism; (5) pneumonia; (6) acute kidney injury (AKI); and (7) re-operation. The above events were recorded by our authors based on the definitions of the 30-day postoperative outcomes patients from the society of thoracic surgeons (STS) national cardiac database (17) and the 2021 ACC/AHA/SCAI guideline for coronary artery revascularization (18).

### Statistical analysis

Statistical analyses were performed using SPSS version 25.0 (IBM Corporation, Armonk, NY). Continuous and categorical variables were shown as mean ± SD and percentages, respectively. Student t test was used to compare continuous variables, and the chi-square test or Fisher exact test was used to compare categorical variables. The variables potentially associated with our endpoints were evaluated using univariate analysis. Variables that had a *p*-value of less than 0.05 in the univariate analysis were further assessed in the multivariate analysis. The power of the association between variables and outcomes was expressed as odds ratio (OR). All statistical tests were two-sided, and differences with *p* < 0.05 were considered to be statistically significant.

## Results

The baseline characteristics of the study population are shown in Table 3. A total of 562 patients met the inclusion criteria for the current study. 63 (11.2%) suffered from severe ACAS. Compared to the non-severe ACAS group, patients with severe ACAS experienced a longer operation time (5.0 ± 1.6 vs. 4.2 ± 1.0, *p* < 0.0001), a higher EuroSCORE (11.0 ± 6.2 vs. 8.3 ± 3.7, *p* < 0.0001), a lower left ventricular ejection fraction (LVEF) (45.6 ± 19.5 vs. 53.7 ± 14.2, *p* < 0.0001), and had a higher prevalence of peripheral arterial disease (50.8% vs. 35.3%, *p* = 0.032) and heart failure (38.0% vs. 23.6%, *p* = 0.041). In addition, severe ACAS was found to be more common in current smokers (41.3% vs. 24.2%, *p* = 0.016).

**Table 3:** Patients’ baseline characteristics.

Variable	Severe ACAS		
	Yes/*n* = 63	No/*n* = 499	*P-value*
Age, mean (SD), y	60.5 (13.6)	62.7 (9.9)	0.216
Sex (male), *n* (%)	35 (55.6%)	289 (57.9%)	0.112
BMI, mean (SD), kg/m^2^	24.9 (3.9)	25.7 (3.3)	0.117
Hypertension, *n* (%)	38 (60.3%)	247 (50.4%)	0.247
Diabetes mellitus, *n* (%)	31 (49.2%)	201 (40.3%)	0.298
Current smoker, *n* (%)	26 (41.3%)	121 (24.2%)	**0**.**016**
LVEF, mean (SD),%	45.6 (19.5)	53.7 (14.2)	**<0**.**0001**
EuroSCORE mean (SD),%	11.0 (6.2)	8.3 (3.7)	**<0**.**0001**
Peripheral arterial disease, *n* (%)	33 (50.8%)	176 (35.3%)	**0**.**032**
COPD, *n* (%)	5 (7.9%)	38 (7.6%)	0.561
Previous MI, *n* (%)	21 (33.3%)	108 (21.6%)	0.122
Previous AF, *n* (%)	10 (15.9%)	34 (6.8%)	0.053
Perioperative IABP use *n* (%)	8 (12.7)	47 (9.4)	0.361
Emergency, *n* (%)	10 (15.9)	44 (8.8)	0.109
Ventricular aneurysm, *n* (%)	2 (3.2)	35 (7.0)	0.140
Duration of operation mean (SD), h	5.0 (1.6)	4.2 (1.0)	**<0**.**0001**
Heart failure, *n* (%)	24(38.0)	118(23.6)	**0**.**041**

ACAS, asymptomatic severe carotid artery stenosis; AF, previous atrial fibrillation; BMI, body mass index; COPD, previous chronic obstructive pulmonary diseases; CVA, cerebral vascular accident; EuroSCORE, European system for cardiac operative risk evaluation; LVEF, left ventricular ejection fraction; MI, myocardial infarction; SD, standard deviation.

Bold value indicates *P*-value less than 0.05, within the 95% confidence interval range.

**Figure 2:**
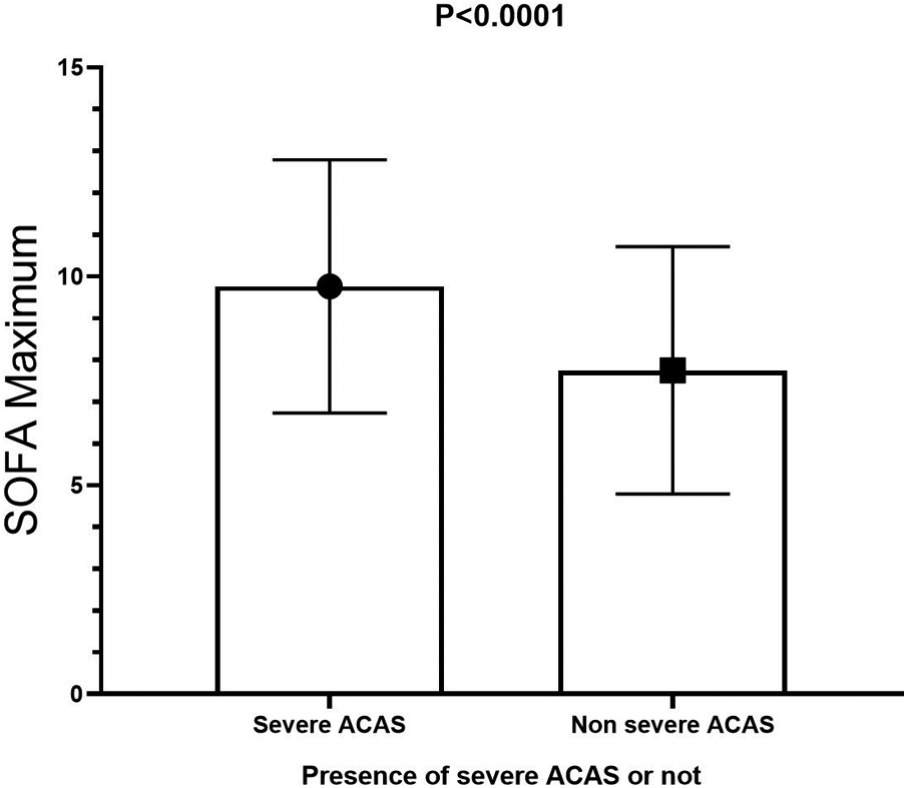
SOFA maximum in the severe ACAS group was significantly higher than that in the non-severe ACAS group. SOFA, sequential organ failure assessment; ACAS, asymptomatic severe carotid artery stenosis.

Short-term postoperative events are summarized in Table 4. The SOFA maximum in the severe ACAS group was significantly higher than that in the non-severe ACAS group **(**9.76 ± 3.03 vs. 7.75 ± 2.96, *p* < 0.0001, (Figure 2), and a higher proportion of patients in the severe ACAS group exhibited severe multi-organ dysfunction (44.4% vs. 14.0%, *p* < 0.0001). In addition, severe ACAS was related to an increased rate of 30-day postoperative MACCEs (15.9% vs. 6.6%, *p* = 0.027), including a 30-day postoperative stroke (9.5% vs. 3.0%, *p* = 0.033). Severe ACAS was associated with an elevated risk of delirium (6.3% vs. 0.8%, *p* = 0.010), and AKI (12.7% vs. 1.6%, *p* < 0.0001). For the other short-term events, there were no significant differences between the two groups.

**Table 4:** Short-term postoperative events.

Short-term postoperative events	Severe ACAS		*P-value*
	Yes/*n* = 63	No/*n* = 499	
Severe multi-organ dysfunction (SOFA maximum ≥11)	28 (44.4%)	70 (14.0%)	**<0**.**0001**
SOFA maximum	9.76 ± 3.03	7.75 ± 2.96	**<0**.**0001**
30-day post MACCEs	10 (15.9%)	33 (6.6%)	**0**.**027**
30-day post mortality	2 (3.2%)	5 (1.0%)	0.209
30-day post stroke	6 (9.5%)	15 (3.0%)	**0**.**033**
30-day post MI	6 (9.5%)	22 (4.4%)	0.116
New-onset AF	23 (36.5%)	141 (28.6%)	0.250
New-onset VA	6 (9.5%)	31 (6.2%)	0.298
Postoperative delirium	4 (6.3%)	4 (0.8%)	**0**.**010**
Pulmonary embolism	0	4 (0.8%)	1.0000
Pneumonia	5 (7.9%)	20 (4.0%)	0.182
AKI	8 (12.7%)	8 (1.6%)	**<0**.**0001**
Re-operation	4 (6.3%)	9(2.0%)	0.063

ACAS, asymptomatic severe carotid artery stenosis; AF, atrial fibrillation; AKI, acute kidney injury; MACCEs, major adverse cardiovascular and cerebral events; MI, myocardial infarction; SOFA, Sequential Organ Failure Assessment; VA, ventricular arrhythmias.

Bold value indicates *P*-value less than 0.05, within the 95% confidence interval range.

The results of the multivariate analysis (Table 5) demonstrated that severe ACAS may be independently associated with severe multi-organ dysfunction (OR, 7.37, 95% CI 4.80–14.30, *p* < 0.0001) after OPCAB. In addition, the higher body mass index (BMI) (OR, 1.35, 95%CI 1.25–1.46, *p* < 0.0001), higher EuroSCORE (OR, 1.07, 95% CI 1.02–1.12, *p*-0.010), peripheral arterial disease (OR, 2.10, 95% CI 1.27–3.48, *p* = 0.004), previous MI (OR, 2.26, 95% CI 1.39–3.69, *p* = 0.001), perioperative IABP use (OR, 3.42, 95% CI 1.74–6.69, *p* < 0.0001), emergency (OR 7.60, 95% CI 3.94–14.36, *p* < 0.0001), ventricular aneurysm (OR, 3.92, 95% CI 1.49–7.70, *p* = 0.004), and longer operating time (OR, 1.32, 95% CI 1.06–1.64, *p* = 0.013) were independent risk factors for severe multi-organ dysfunction. On the contrary, higher preoperative LVEF may be independently related to a decreased incidence of severe multi-organ dysfunction.

**Table 5:** Independent predictors of severe multi-organ dysfunction (SOFA maximum ≥11).

Variable	Multivariate analysis OR (95% CI)	*P*-value
Severe ACAS	**7.37** (**4.80–14.30)**	**<0**.**0001**
Age	1.01 (0.99–1.04)	0.473
Sex (male)	1.19 (0.72–1.98)	0.503
BMI	**1.35** (**1.25–1.46)**	**<0**.**0001**
Hypertension	1.50 (0.92–2.42)	0.099
Diabetes mellitus	1.08 (0.68–1.72)	0.759
Current smoker	1.21 (0.74–1.96)	0.450
LVEF	**0.98** (**0.96–0.99)**	**0**.**009**
EuroSCORE	**1.07** (**1.02–1.12)**	**0**.**010**
Peripheral arterial disease	**2.10** (**1.27–3.48)**	**0**.**004**
COPD	0.99 (0.41–2.38)	0.977
Previous MI	**2.26** (**1.39–3.69)**	**0**.**001**
Previous AF	0.65 (0.28–1.64)	0.360
Perioperative IABP use	**3.42** (**1.74–6.69)**	**<0**.**0001**
Emergency	**7.60** (**3.94–14.36)**	**<0**.**0001**
Ventricular aneurysm	**3.92** (**1.49–7.70)**	**0**.**004**
Duration of operation	**1.32** (**1.06–1.64)**	**0**.**013**
Heart failure	1.23 (0.71–2.15)	0.460

SOFA, sequential organ failure assessment; ACAS, asymptomatic severe carotid artery stenosis; AF, previous atrial fibrillation; BMI, body mass index; COPD, previous chronic obstructive pulmonary diseases; CVA, cerebral vascular accident; EuroSCORE, European system for cardiac operative risk evaluation; LVEF, left ventricular ejection fraction; MI, myocardial infarction; SD, standard deviation.

Bold value indicates *P*-value less than 0.05, within the 95% confidence interval range.

Based on the results of multivariate analysis (Table 6), the current research also demonstrated severe ACAS may be independently associated with 30-day postoperative stroke (OR, 2.83, 95% CI 1.03–7.75, *p* = 0,043). The male sex (OR 2.68, 95% CI 1.08–6.62, *p* = 0.033), emergency (OR, 10.67, 95% CI 3.62–31.40, *p* < 0.0001), and longer operating time (OR, 1.86, 95% CI 1.30–2.65, *p* = 0.001) were independent risk factors for 30-day postoperative stroke.

**Table 6:** Independent predictors of 30-day postoperative stroke.

Variable	Multivariate analysis OR (95% CI)	*P*-value
Severe ACAS	**2.83** (**1.03–7.75)**	**0**.**043**
Age	1.01 (0.97–1.05)	0.691
Sex (male)	**2.68** (**1.08–6.62)**	**0**.**033**
BMI	1.03 (0.91–1.17)	0.651
Hypertension	1.50 (0.57–3.97)	0.416
Diabetes mellitus	0.49 (0.18–1.31)	0.156
Current smoker	0.34 (0.10–1.12)	0.092
LVEF	1.00 (0.97–1.03)	0.887
EuroSCORE	1.03 (0.94–1.13)	0.323
Peripheral arterial disease	0.44 (0.18–1.10)	0.079
Previous MI	0.35 (0.09–1.32)	0.121
Previous AF	0.53 (0.09–3.23)	0.494
Emergency	**10.67** (**3.62–31.40)**	**<0**.**0001**
Ventricular aneurysm	1.70 (0.21–14.07)	0.622
Duration of operation	**1.86** (**1.30–2.65)**	**0**.**001**
Heart failure	0.27 (0.06–1.29)	0.100

ACAS, asymptomatic severe carotid artery stenosis; AF, previous atrial fibrillation; BMI, body mass index; EuroSCORE, European system for cardiac operative risk evaluation; LVEF, left ventricular ejection fraction; MI, myocardial infarction; SD, standard deviation.

Bold value indicates *P*-value less than 0.05, within the 95% confidence interval range.

## Discussion

To the best of our knowledge, this is the first single-center, retrospective observational study that demonstrate the correlation between severe ACAS and the incidence of multi-organ dysfunction after OPCAB. The key results of this study are that SOFA maximum in the severe ACAS group was significantly higher than that in the non-severe ACAS group, and the severe ACAS was independently associated with severe multi-organ dysfunction after OPCAB, which may be associated further with an increased rate of 30-day postoperative mortality and complications. In addition, we demonstrated that severe ACAS may be independently associated with 30-day stroke after OPCAB.

Although severe ACAS is associated with an increased risk of stroke and the European Society of Cardiology/American Heart Association guidelines classify individuals with severe ACAS as high-risk for MACCEs prevention, its potential impact on the 30-day adverse events after OPCAB remains incomprehensible. For the 30-day postoperative cerebral events, Santarpino et al. found that ACAS ≥ 90% was independently associated with 30-day stroke after OPCAB (4). However, Preop screening of CAS is routine in CABG and OPCAB, thus ACAS will be seen (4). On the contrary, Mahmoudi et al. found that severe ACAS is not an independent predictor for the 30-day postoperative stroke in patients undergoing OPCAB, and pre-operative screening for ACAS prior to OPCAB should be personalized and based on clinical discretion (19). A meta-analysis focusing on ACAS individuals revealed that patients with bilateral ACAS ≥ 50% had a stroke risk of 6.5% following cardiac surgery (20). No significant differences in the incidence of 30-day stroke after OPCAB between patients with and without severe ACAS had also been reported in other studies (21–23). In addition, at present there is a paucity of clinical studies examining the association between severe ACAS and 30-day postoperative mortality, as well as other organ-related events. In summary, firstly we thought the inconsistent criteria utilized for evaluating severe ACAS may contribute to the discrepant findings reported in the previous literature. There is a lack of literature regarding the definition of severe ACAS using the criteria recommended by current guidelines. Secondly, the limited sample size precludes directed the exploration of the independent correlation between severe ACAS and 30-day postoperative mortality in patients undergoing OPCAB. Based on the limited data available, it is imperative to employ a novel primary endpoint that makes it possible to investigate the association between severe ACAS and 30-day mortality, as well as complications after OPCAB.

As a robust predictor of 30-day outcomes following cardiac surgery, severe multi-organ dysfunction demonstrates superior accuracy, specificity, and independence from treatment when forecasting patient outcomes (14, 24). Four scoring systems were used to define multi-organ dysfunction: the Glasgow Coma Scale, poison severity score, Multiple Organ Dysfunction Score (MODS), and SOFA score. The SOFA score was adopted based on the following advantages: (1) A maximum SOFA score ≥11, namely severe multi-organ dysfunction, is the gold standard for evaluating 30-day mortality after OPCAB with a sensitivity of 95% (13); (2) the SOFA score is more feasible with cardiovascular and cerebrovascular function evaluation (24). The current research regarded the severe multi-organ dysfunction instead of the 30-day postoperative mortality as the first endpoint.

The potential adverse effects of severe ACAS on the 30-day postoperative mortality and organ-related complications may be attributed to the following factors. First, in severe ACAS patients undergoing OPCAB, the occurrence of 30-day cerebral events may be ascribed to the impairment in cerebral hemodynamics. Preexisting hemodynamic impairments serve as a key predictive factor for the occurrence of stroke/TIA (25). Severe ACAS, especially asymptomatic carotid artery occlusion, can lead to not only a reduction in the perfusion pressure on the ipsilateral side but a recruitment of secondary collaterals, both of which may be associated with hemodynamic impairment (3, 26). And the presence of hemodynamic impairment in both hemispheres has been reported in asymptomatic individuals with carotid artery occlusion. Secondly, the rupture of the severe ACAS that was associated with atherosclerotic plaque can result in distal embolism and subsequent 30-day postoperative mortality and stroke/TIA (3). Thirdly, atherosclerosis of severe ACAS is another significant cause of 30-day adverse outcomes after OPCAB (27). Kanemitsu et al. confirmed that the prevalence of complications was significantly higher in individuals with severe atherosclerosis who underwent OPCAB, compared to those who without severe atherosclerosis (28).

Whether severe ACAS patients will lead to a clinically meaningful benefit from a OPCAB combined carotid endarterectomy remains to be investigated further. Mahmoudi et al. identified that severe ACAS may lead to the development of 30-day postoperative adverse outcomes, but its role is unlikely to be a critical one in severe ACAS patients undergoing OPCAB. Therefore, they opposed the customary employment of OPCAB with carotid endarterectomy (19). In the Coronary Artery Bypass Graft Surgery in Patients with Asymptomatic Carotid Stenosis Study (CABACS), the authors compared with OPCAB alone, severe ACAS patients undergoing OPCAB with carotid endarterectomy had a similar five-year risk of mortality and stroke (29). They recommended OPCAB alone for severe ACAS individuals (29). Our findings supported personalized surgical management based on refined clinical evaluation for severe ACAS patients undergoing OPCAB. Since our results confirmed that severe ACAS was independently associated with severe multi-organ dysfunction after OPCAB, a prompt intervention for severe ACAS may improve the prognosis of patients undergoing OPCAB theoretically (RCTs verification required). However, it has been reported that the combination of such an intervention with OPCAB is associated with a higher incidence of adverse postoperative outcomes (1, 30). Therefore, the potential benefits and risks of OPCAB with carotid endarterectomy should be assessed and personalized. In addition, the role of multi-organ parameters, especially the cardio-cerebral factors, should be emphasized during the process of severe ACAS management. For the etiology of 30-day mortality or stroke, the North American Symptomatic Carotid Endarterectomy Trial (NASCET) (27) demonstrated that strokes of cardioembolic origins among the severe ACAS patients was probably underestimated and could not be prevented by carotid endarterectomy. And as discussed above, severe cardio-cerebral atherosclerosis is another significant cause of 30-day adverse outcomes after OPCAB.

It is worth mentioning that the current study demonstrated a longer operation time was associated with severe multi-organ dysfunction and an increased rate of 30-day MACCEs. Our thinking on this issue is that patients with ACAS have increased risk factors before surgery, such as more severe atherosclerosis and ischemic damage to brain blood flow. In addition, we also observed that the degree and number of coronary artery stenosis may be more complex in patients with ACAS. Therefore, the anesthesia time and surgical plan may be adjusted, and the final time may be longer. Previous study confirmed that a longer operation time is an independent predictor of longer ICU stay, regardless of factors such as the number of blood products transfused, CPB/myocardial ischemic times, and ejection fraction, which are indirect markers to assess the complexity level of OPCAB procedures (31). However, prospective studies are still needed to directly demonstrate whether the association between surgical duration and adverse outcomes is affected by the complexity of cardiovascular disease and OPCAB procedures.

Our study has some limitations. First, the results lack long-term follow-up. Therefore the findings are limited to the 30-day timeframe and cannot be extrapolated beyond it. In addition, it is a small, single-center, retrospective observational study. High quality, large-scale, and multicenter randomized controlled trials are required to further confirm the optimal management of severe ACAS patients undergoing OPCAB. In addition, the explanation of adverse outcomes in the presence of ACAS is multifactorial. For instance, a valuable study demonstrated the risk of 30-day MACCEs was significantly greater in patients with diabetes, without clinical cardiovascular disease, who have both a longer diabetes duration and significant ACAS, compared with those who with a shorter duration and/or nonsignificant ACAS (32). And the confounding factor, namely diabetes, has been eliminated through multivariate analysis in the current study. However, since this is a retrospective observational study, the possibility of some unmeasured confounding variables still cannot be ruled out.

## Conclusions

Severe ACAS was independently associated with severe multi-organ dysfunction, measured by SOFA maximum ≥11 after OPCAB, which may be associated further with an increased rate of 30-day postoperative mortality and complications. Our data contributes to the growing evidence that highlights the importance of personalized assessment for potential advantages and disadvantages in prognosis of severe ACAS patients undergoing OPCAB with carotid endarterectomy. Nevertheless, the role of multi-organ parameters, especially cardio-cerebral factors, should be emphasized during the process of severe ACAS management.

## Data Availability

The raw data supporting the conclusions of this article will be made available by the authors, without undue reservation.
